# Mapping Evidence on Child-Focused Interventions for Promoting Healthy Sleep Habits: A Scoping Review

**DOI:** 10.3390/clockssleep8020017

**Published:** 2026-04-07

**Authors:** Ana Patrícia Dias, Fernanda Loureiro

**Affiliations:** 1 Cinesitraquinas—Clínica Pediátrica e da Família, 1649-023 Carcavelos, Portugal; apatriciamdias@gmail.com; 2 Universidade Católica Portuguesa, Faculdade de Ciências da Saúde e Enfermagem, Centro de Investigação Interdisciplinar em Saúde (CIIS), 1649-023 Lisboa, Portugal

**Keywords:** sleep, sleep hygiene, child, health promotion, patient participation, health education, review literature

## Abstract

Sleep is critical for physical growth and healthy child development. Traditionally, interventions targeting sleep improvement in children have focused on the parents. This scoping review aimed to identify and summarize the available evidence on child-focused interventions designed to promote healthy sleep habits among children aged 3–12 in healthcare settings. The review was conducted in accordance with JBI guidelines. A comprehensive search strategy was employed, encompassing databases such as PubMed, CINAHL, Psychology & Behavioral Sciences Collection, Education Source, Scopus, Web of Science, the Public Health Database, and Portugal’s Open Access Scientific Repository. Following identification and screening, 15 articles were included. Three types of interventions were identified: isolated, combined, and structured programs. Overall, the studies suggest that, while sleep-related knowledge tends to improve, achieving sustained, long-term behavioral change remains challenging in this age group. Active child participation appears essential for promoting lasting results and developing more tailored, child-friendly interventions.

## 1. Introduction

Sleep is not simply the absence of wakefulness; instead, it is a complex and active neurophysiological process that is vital for survival and a fundamental function of the developing brain [1]. According to The American Academy of Sleep Medicine [2], healthy sleep requires adequate quantity, proper timing, good quality, regularity, and the absence of sleep disturbances. Sleep is a critical determinant of physical growth and healthy child development, and insufficient sleep or compromised sleep quality contributes to adverse physical and mental health consequences [3].

Despite their fundamental role, sleep habits in childhood are often compromised. There has been an increase in the prevalence of sleep problems among children in recent years [4,5], and there is growing evidence that these difficulties are linked to disparities in child and adolescent development [4,6]. These findings emphasis the importance of systematically screening for sleep disorders during pediatric consultations and highlight the need to prioritize targeted health promotion and preventive interventions in this area.

Interventions aimed at improving infant sleep have several limitations. These include the frequent absence of the child as an active participant in the behavioral change process [7,8]. Despite evidence indicating that interventions in childhood are most effective when they incorporate direct educational experiences with children rather than relying exclusively on parental training [9], this approach persists.

As most existing reviews focus on interventions in which children do not play an active role, there is clearly a need for a synthesis that prioritizes interventions specifically designed to promote healthy sleep habits by involving children directly. For the purposes of this review, we focused on pre-school children (aged 3 to 5 years) and school-aged children (aged 6 to 12 years). These age ranges were selected because children demonstrate increasing levels of independence, initiative, and self-regulatory capacity from the pre-school years onwards, all of which are essential for meaningful engagement in health-promoting behaviors [9].

To clarify the main interventions available in the literature, a review was conducted. We choose to perform a scoping review as it is the preferred approach when the aim is to map existing evidence and clarify key concepts and definitions within a specific field [10]. Therefore, this review aims to identify and summarize the available evidence on child-focused interventions designed to promote healthy sleep habits among children (3–12 years) within healthcare settings. The guiding research question was: What interventions aimed at preschool and school-aged children are available to promote healthy sleep habits in healthcare settings?

## 2. Materials and Methods

Before conducting this review, we searched several public registration platforms, including the Open Science Framework (Charlottesville, Virginia, United States of America), International prospective register of systematic reviews—PROSPERO (York, United Kingdom), Figshare (London, United Kingdom), International Platform of Registered Systematic Review and Meta-Analysis Protocols—Inplasy (Middletown, United States of America), the Cochrane Library of Systematic Reviews (London, United Kingdom) and the Joanna Briggs (JBI) Institute Systematic Review Register (Adelaide, South Australia, Australia), to identify any literature reviews on this topic that were either ongoing or had been completed. No reviews were found that focus specifically on interventions to promote healthy sleep directed to children. This indicates a clear knowledge gap, which this review aims to address. We used the JBI guidelines for Scoping Reviews [11].

### 2.1. Protocol and Registration

This review’s protocol was initially developed in January 2025 and subsequently registered on the Open Science Framework (OSF). The full protocol, including detailed methodology and planned analytical procedures, is publicly available on OSF under the identifier 10.17605/OSF.IO/FDPGZ and can be accessed at https://doi.org/10.17605/OSF.IO/FDPGZ registered on 21 February 2025.

### 2.2. Eligibility Criteria

In line with the recommendations for scoping reviews, we used the PCC (Participants, Concept, Context) framework to inform the eligibility criteria.

Regarding participants, we included studies involving pre-school children (aged 3–5) and school-age children (aged 6–12). We also considered studies that implemented joint interventions with parents and pre-school and/or school-age children, as well as studies that included broader age ranges of children, provided that the results relating to pre-school and/or school-age children could be clearly identified. Regarding the concept, we included studies examining interventions aimed at promoting healthy sleep among pre-school and school-age children. Finally, in context, we considered studies conducted in any health-related context, whether hospital or community-based, and included children who were either healthy or living with an illness to ensure a comprehensive and inclusive scope.

We excluded the following types of study: secondary research (e.g., literature reviews); letters to the editor; book reviews; opinion pieces; and articles involving populations outside the defined age range, or in which the intervention effects for this age group could not be isolated. Studies focusing on children with neurodevelopmental disorders were also excluded because their sleep patterns present distinct characteristics, making it more appropriate to consider them separately from the general population.

### 2.3. Information Sources

We used the following databases: PubMed, CINAHL, the Psychology & Behavioural Sciences Collection, Education Source, Scopus, Web of Science, Public Health Database for grey literature, and Portugal’s Open Access Scientific Repository. When an article was unavailable, we contacted the authors directly to retrieve it. The search was performed on 10 July 2025.

### 2.4. Search

The search strategy was conducted in three stages, as recommended by the JBI [11]. First, a search was conducted in the PubMed and EBSCO databases to identify relevant keywords from the titles, abstracts, and descriptors of articles. This step was taken to identify the most suitable keywords for the search strategy.

In the second stage, an experienced librarian was consulted to help formulate the search equation using MeSH descriptors and perform the search. Depending on the database, the search equation included key terms combined using Boolean operators and entered into their respective search engines.

In the third stage, the bibliographic references of the identified articles were analyzed to locate further studies.

### 2.5. Selection of Sources of Evidence

This review included studies that were first screened through an analysis of titles and abstracts. All retrieved records were exported to Rayyan^®^ software (version 1.6.1), where duplicate entries were removed. Two reviewers then independently assessed the titles and abstracts. Any disagreements were resolved through discussion; when consensus could not be reached, a third reviewer was consulted. Articles considered eligible at this stage were then screened according to the predefined inclusion criteria.

To ensure comprehensive identification of available evidence, no restrictions were applied with regard to the year of publication. Eligible studies were limited to full-text articles.

### 2.6. Data Charting Process

In this phase, the full texts of the included articles were read in detail, and relevant information was systematically extracted using a data-charting form that had been developed previously. Once the data had been extracted, the reviewers compared their charted data to ensure accuracy and consistency. Any discrepancies identified were discussed and resolved through consensus. This was done to enhance data-charting reliability.

### 2.7. Data Items

The data extracted from each article was aligned with the aim of this scoping review, focusing on key variables relevant to understanding interventions that promote healthy sleep in pre-school and school-age children. The data items included the following: the study’s objective, its methodology, the authors’ main conclusions or reported outcomes and the intervention. We used the JBI Level of Evidence tool [12] to characterize the type of study methodology.

### 2.8. Synthesis of Results

To summarize the findings, we followed a narrative approach, in line with the latest guidance on scoping reviews. Following data extraction, the reviewers organized the results into broad dimensions or thematic categories reflecting recurrent patterns across the included studies. This involved identifying similarities and differences in the aims, characteristics of the interventions, and reported outcomes. The thematic grouping made it possible to present the evidence in a structured way, which in turn made it easier to understand the types of interventions that were used.

## 3. Results

### 3.1. Selection of Sources of Evidence

A comprehensive search was conducted in the previously identified databases. From our initial sample of 1662 articles, 89 were initially selected based on their titles and abstracts. Thirteen of these articles were included in this review after the full text was read. An additional two articles were identified through manual screening of the reference lists of included studies, resulting in a final sample of 15 articles.

No language restrictions were applied during the initial search. However, following the screening process, only articles published in English, Portuguese or Spanish were retained, since these are the languages in which the review team is fluent. Four articles published in other languages were identified, and the corresponding authors were contacted to ask if versions in one of the eligible languages were available. However, no such versions were available. The authors were also contacted when full-text articles could not be accessed through institutional resources. Despite these efforts, six articles could not be retrieved and were therefore excluded.

**Figure 1:**
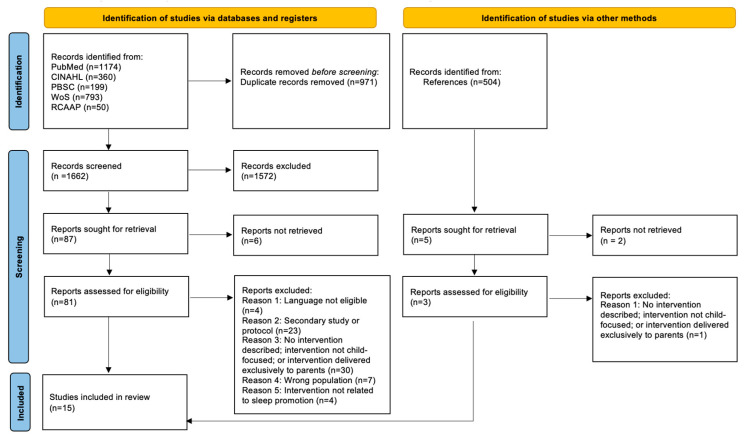
PRISMA flowchart for study selection.

Secondary studies, including literature reviews and study protocols, were excluded as they did not report primary empirical results relevant to the review objectives. Thirty articles were excluded because the intervention was not described, was not child-focused, or was delivered exclusively to parents, even when outcomes were measured in children. A further seven studies were excluded due to criteria relating to the population, either because the sample fell outside the predefined age range or because results for the target population could not be isolated. Finally, studies were excluded if sleep was assessed only as a secondary outcome of interventions that primarily targeted other conditions (e.g., obesity). Figure 1 presents the PRISMA flow diagram summarizing the study selection process.

### 3.2. Characteristics of Sources of Evidence

The interventions were identified and classified into three groups: isolated interventions, combined interventions and programs.

Isolated interventions were found in articles that studied only the effectiveness or impact of a single intervention, such as games [13,14], animated videos [15], videos on sleep hygiene [16], or yoga [17]. Combined interventions were found in articles that studied the effectiveness or impact of two or more interventions [18,19]. Programs were the most prevalent type of intervention found in this review. These consisted of several structured, organized, and continuous interventions over a period of time [20,21,22,23,24,25].

### 3.3. Results of Individual Sources of Evidence

We extracted the relevant data from each article retrieved, which is summarized in Table 1.

### 3.4. Synthesis of Results

#### 3.4.1. General Characteristics

We analyzed studies published between 2011 and 2025. All of the studies were quantitative in nature. The majority of these studies evaluated the effectiveness of intervention programs.

Studies were from a variety of geographical contexts, including Australia [20,26], the United States [14,15,17,22,23,25,27], Japan [19], the United Kingdom [16,18], Brazil [13], Iran [24], and Spain [21]. Methodologically, the articles range from randomized controlled trials, classified as JBI Level 1.c evidence, to quasi-experimental studies, case reports, and prospective cohorts. Overall, the studies converge on the idea that, although knowledge about sleep is steadily improving, long-term (6 to 12 months) behavior change remains a complex challenge in this age group [13,18,20,22].

#### 3.4.2. Children Age Groups

In the younger group (3 to 5 years), children still have very decreased participation. Interventions predominantly focus on the direct involvement of caregivers and the structuring of domestic routines [23,27]. Personalized programs, such as ‘Sleep Well!’, have demonstrated that a combination of parental education and flexible strategies can significantly reduce the use of electronics in the bedroom, sleep latency and the duration of night-time awakenings [23]. Implementing calming routines, such as doing yoga before bedtime, has proven to be an effective strategy for improving perceived sleep quality and reducing family chaos [17]. Additionally, brief interventions in the first year of school accelerate the resolution of moderate to severe sleep problems, resulting in medium- and long-term (6 to 12 months) improvements in children’s prosocial behavior [26]. Cognitive behavioral play therapy has also shown promise in reducing anxiety and night-time fears in this age group [14].

Interventions for children 6 to 9 years old tend to focus on environmental modifications and behavioral stabilization, often involving high levels of interactive engagement and participation. In Almondes et al.’s [13] study, children in this age group showed an immediate reduction in the number of electronic devices in their bedrooms and reported performing fewer actions with these devices after participating in “serious games” like Perfect Bedroom. However, these changes were not sustained at a one-month follow-up. The use of animated educational media was successful in decreasing bedtime resistance, sleep anxiety, and night awakenings [15]. In elite athletic contexts [21], children showed significant improvements in academic performance following personalized sleep education. Paradoxically, this same group experienced a decline in sleep quality and mood during the sports season, possibly due to increased competitive pressure and a lack of tools to manage high physiological activation.

For older children, aged 10 to 12 years, interventions increasingly target participation, internal motivation, self-efficacy, and the cognitive link between sleep and performance. The introduction of short, animated sleep hygiene videos resulted in significant improvements in sleep duration, sleep latency, and sleep efficiency for 5th and 6th graders [16]. Several universal programs for this age range reported a sustained increase in sleep knowledge, but this knowledge frequently failed to translate into robust behavioral changes [18]. The Sleep to Enhance Educational Performance in Schools (SLEEPS) program, based on the Theory of Planned Behavior, led to significant improvements in sleep-related beliefs and self-efficacy, as well as reductions in daytime sleepiness and internalizing symptoms (such as sadness or anxiety) [22]. When children in grades 4–6 were allowed to select their own target behaviors and monitor them via a sleep diary, they successfully advanced their bedtimes and increased their sleep duration [19]. Unlike the younger group, older athletes showed significant improvements in both sleep quality and academic performance following personalized interventions [21]. Researchers suggest this is because older pre-adolescents have the mental maturity to recognize the direct link between recovery behaviors (sleep) and their performance outcomes.

#### 3.4.3. Gender

Regarding gender, it is primarily discussed as a demographic characteristic or a controlled variable, with most studies reporting no significant differences in how boys and girls respond to sleep interventions. However, a few specific differences and trends were noted.

Intervention efficacy by gender. In Rigney et al. [20] study’s, subgroup analysis revealed that boys responded significantly better than girls to the intervention regarding objective time spent in bed; in a Japanese study on self-help treatment found that 4th-grade girls showed a larger effect size in sleep duration compared to boys of the same grade [19]. A personalized athletic program in Spain explicitly reported that gender did not moderate outcomes or show significant differences in sleep quality, duration, or academic improvements [21].

Baseline behaviors and knowledge. One study noted that only 37.8% of children correctly identified whether boys or girls typically have “deeper sleep” [18]. The literature cited within the articles found in this review suggests that, at baseline, boys may have shorter sleep durations on non-school nights, while girls may sleep less in certain minority populations.

Caregiver participation. There is a heavy gender imbalance among participating caregivers, with 92% to 100% of involved adults being mothers [14,17,23,27]. This limits the evidence regarding the role of fathers in implementing these routines.

## 4. Discussion

### 4.1. Synthesis of Results

In this review, we identified three main types of interventions that promote sleep in children ages 3 to 12 years: isolated interventions, combined interventions, and programs.

Regarding isolated interventions, characterized by the application of a single therapeutic tool or therapy, although they have been shown to be effective in immediately improving specific symptoms, their long-term (6 to 12 months) stability may be limited [24]. In fact, isolated interventions targeting sleep have been found to be useful in specific populations and contexts [28]. Educational audiovisual resources, such as sleep hygiene videos or animated films, have been shown to reduce resistance to bedtime, improve latency and increase sleep duration in school-aged children [15,16]. The educational value of videos and other digital resources is widely recognized. However, according to current sleep hygiene recommendations for children, screen time should be avoided during the hour before bedtime. Screen exposure, particularly to blue light and stimulating content, may negatively affect sleep onset, duration, and quality [29]. Clinical approaches such as reflexology, while reducing insomnia in the post-test period, have demonstrated that symptoms tend to reappear after a two-month follow-up period [24]. These results suggest that single-component interventions may be useful for raising awareness or providing symptomatic relief, but they may lack the mechanisms needed to maintain behavioral change.

Interventions that combine two to four strategies, such as consultations, technological tools, and playful activities, focus primarily on modifying routines and the home environment [23,27]. Strategies integrating brief consultations with sleep management plans and informational brochures resulted in faster sleep problem resolution and long-term (6 to 12 months) gains in prosocial behavior [26]. Similarly, coupling sleep hygiene with additional behavioral strategies, such as mindfulness, yields better outcomes than sleep hygiene alone [30]. Using serious games combined with activity mapping immediately reduced electronics use in bedrooms and combining yoga routines with parental support improved sleep quality and reduced domestic chaos [13,17]. In fact, the use of combined strategies that integrate interaction between parents and children has been found useful in promoting healthy habits among children [31]. Families have responded positively to the use of complementary technologies, such as text messages and infographics, which promote adherence to habits such as reducing caffeine consumption and removing screens from bedrooms [23,25].

Multicomponent programs that are sequentially implemented and embedded within school curricula or sports settings appear to produce the most robust and sustained effects on children’s sleep-related knowledge, beliefs, and behaviors [18,22]. Unlike single-session or information-based approaches, these interventions combine educational, behavioral, and environmental components, thereby addressing multiple determinants of sleep health. Programs such as ACES (Australian Centre for Education in Sleep) and SLEEPS integrate classroom-based instruction, interactive group activities, and parent-focused sessions grounded in the Theory of Planned Behavior [18,22]. By targeting attitudes, perceived behavioral control, and social norms, these initiatives have demonstrated consistent improvements in children’s sleep knowledge, self-efficacy, and attitudes toward adopting healthy sleep practices. Importantly, the inclusion of parental involvement strengthens the home–school connection and facilitates the translation of knowledge into daily routines [32,33].

At the clinical level, intensive Cognitive Behavioral Therapy for Insomnia programs, such as KiSS (Kinder mit Schlafstörungen Programm), further illustrate the added value of comprehensive, structured approaches [24]. These interventions typically combine psychoeducation, stimulus control, sleep restriction, relaxation strategies, and cognitive restructuring. Evidence indicates that such multicomponent programs are more effective than isolated or single-strategy interventions, producing clinically meaningful and sustained reductions in insomnia symptoms at follow-up [34,35].

In high-performance or elite sports contexts, similarly integrated programs have been applied with positive outcomes [21]. Programs that incorporate individualized sleep counseling, structured family sessions, and training for coaches and technical staff have demonstrated improvements not only in sleep duration and quality but also in daytime functioning and academic performance among young athletes [36]. These findings suggest that addressing sleep within a broader ecological framework, engaging children, families, educators, and relevant professionals, enhances both behavioral adherence and long-term (6 to 12 months) impact.

Overall, the evidence supports the implementation of coordinated, multilevel strategies that extend beyond isolated sleep hygiene advice. By simultaneously targeting individual knowledge, family practices, and contextual influences, multicomponent interventions appear better positioned to produce meaningful and sustainable gains in children’s sleep health.

Articles identified in this review suggest that positioning children as active participants, rather than passive recipients of parental rules, is a critical factor in the efficacy of sleep interventions [13,18]. While universal education programs successfully improve knowledge, the transition to lasting behavioral change often depends on the child’s internal motivation, perceived self-efficacy, and direct engagement with the intervention materials [13,18,22]. Children’s participation has been found to be essential for engaging children as active participants and for improving health outcomes [37,38,39].

We were able to identify central themes that highlight the relationship between child participation and intervention success.

1. Building Self-Efficacy through Choice and Self-Monitoring

Interventions that empower children to take ownership of their habits show significant behavioral improvements. For example, in a study of Japanese elementary students, children selected one specific behavior to change and monitored it using a sleep diary [19]. This active choice, supported by self-monitoring, led to significant advancements in bedtime and increased sleep duration. Similarly, the SLEEPS program used motivational interviewing to target self-efficacy, recognizing that a child’s belief in their own control over their sleep is a stronger predictor of change than simple knowledge [22].

2. Developmental Appropriateness and Play-Based Engagement

For younger children, participation appears to be more effective when delivered through developmentally appropriate modes that reduce anxiety and build coping skills. By playing out stories with dolls to solve sleep-related problems (like fear of the dark or separation), children achieve mastery over their environment, leading to reduced bedtime resistance and anxiety [14]. Tools like the “Perfect Bedroom” game or animated movies facilitate high engagement rates by making the learning process fun and interactive [13,15]. These methods have been shown to immediately reduce the presence of electronics in the bedroom and improve sleep quality [13,15]. The KiSS program uses a therapy puppet to teach relaxation and imaginative techniques, using rewards and certificates to maintain the child’s motivation even after the sessions end [24].

3. Awareness of the Recovery-Performance Link

The efficacy of participation is also linked to the child’s maturational age [40]. Research with athletes indicates that older participants showed the most significant improvements in sleep quantity and quality because they were more aware of the direct relationship between recovery behaviors and their athletic and academic performance [21]. In contrast, younger athletes who lacked these personalized tools for self-regulation experienced a decline in sleep quality, highlighting the need for interventions to equip younger children with tools to lower physiological activation and manage overthinking [21].

4. Co-Creation and Cultural Humility

Modern interventions emphasize co-productive processes where children, caregivers, and teachers collaborate on the program’s design [22]. Recent evidence underscores that involving children as active collaborators, where their insights and lived experience are explicitly valued in intervention development, enhances the relevance and acceptability of the resulting programs. Systematic evidence from co-design research with children indicates that interventions shaped through participatory methodologies are more aligned with participants’ preferences, a factor strongly associated with greater satisfaction and engagement with the strategies developed [41,42]. When children feel their expertise on their own life is appreciated, a core tenet of cultural humility, they report higher satisfaction and are more likely to use the strategies in the future. This collaborative approach allows for flexibility, such as when a family shifts goals from independent sleep to safer co-sleeping based on their specific needs and preferences [23].

In summary, the sources indicate that while parental support is necessary to structure the environment, the child’s active buy-in is the engine of behavioral change [13,16,22]. Engaging children through play, choice, and performance-related motivation transforms sleep hygiene from a set of enforced rules into a self-regulated health behavior [16,19,21]. Since all included studies involved some degree of child participation, our findings highlight its consistent presence rather than demonstrating the comparative effectiveness of different levels of participation.

The evolution of studies between 2011 and 2025 reveals a transition from purely educational models to approaches based on planned behavior theory and the use of technology. Although total sleep duration is difficult to extend permanently through universal interventions, improvements in sleep quality and routine stability have shown consistent collateral benefits in mood, mental acuity, and reduction in internalization symptoms, underscoring the importance of integrating sleep education into the school curriculum and primary health care.

### 4.2. Limitations

As with any research, several limitations must be acknowledged. Most of the included studies relied on small, relatively homogeneous samples; used subjective measures; lacked control groups; had limited statistical power; and included only short follow-up periods. These factors constrain the robustness and generalizability of our analysis. Although we targeted children aged 3–12 years specifically, some studies in this range did not report age-stratified results, which made it difficult to draw age-specific conclusions. The lack of research comparing different levels of child participation makes it difficult to draw definitive conclusions about its relative impact. Finally, as with any review, the methodological choices made in our protocol, such as the selection of databases and search terms, may have limited the scope of the included evidence and consequently our analysis.

## 5. Conclusions

We identified three main types of interventions: isolated, combined, and broader structured programs. Consistently, studies across these categories indicate that, while sleep knowledge tends to improve following an intervention, translating this knowledge into sustained behavioral change remains a complex challenge for children aged 3–12.

While improvements in short-term outcomes are often observed, such as increased awareness of healthy sleep practices, shifts in bedtime routines, and temporary gains in sleep duration, these effects frequently attenuate over time without continued reinforcement. Studies emphasize that knowledge acquisition alone is insufficient to modify the entrenched family routines, environmental factors, and developmental influences that shape children’s sleep patterns. They highlight the importance of multi-level approaches that actively engage both children and caregivers, incorporate opportunities for skill practice, and include follow-up or booster sessions to enhance sustainability. Overall, the evidence suggests that future interventions should shift from merely delivering information to adopting participatory, developmentally tailored, and context-sensitive strategies to promote long-term (6 to 12 months) behavioral change.

Evidence from this review also highlights that for sleep interventions to be truly effective, children must be positioned as active participants rather than passive recipients of parental rules. A significant limitation of traditional programs is that they often rely solely on information provided to or by parents, whereas modern evidence suggests that engaging children directly in the change process is essential for building internal motivation and self-efficacy. Interventions that utilize “serious games,” interactive animated videos, and child-led mapping strategies allow students to evaluate their own sleep environment and pre-sleep activities. When children are empowered to select their own target behaviors and monitor them through self-help tools like sleep diaries, they develop a sense of mastery and responsibility that is more likely to lead to immediate improvements in bedtime resistance and sleep habits.

This active participation is developmentally crucial as children grow and begin to take more control over their own behavioral habits. For younger age groups, this participation is successfully fostered through developmentally appropriate modes such as cognitive-behavioral play, where children model coping solutions with puppets, or through interactive yoga routines that help them regulate their own arousal. By involving children in co-creative learning and motivational interviewing, interventions can shift the focus from simple knowledge acquisition to the cultivation of self-regulating and responsible behaviors. Sources indicate that while parental support is necessary to modify the physical environment, it is the child’s active buy-in and perceived expertise in their own life that drives sustained improvements in psychosocial health and academic enablers.

Future research should prioritize larger and more diverse samples, longer follow-up periods, and participatory, developmentally tailored interventions to enhance the sustainability and real-world impact of sleep promotion strategies.

## Figures and Tables

**Table 1:** Results extracted from individual sources.

Author, Year	Objective	Type of Study	Main Conclusions	Intervention
Quach et al., 2011 [26]	To determine the feasibility of screening for childhood sleep problems and the effectiveness of a brief behavioral intervention.	RCT	The intervention resulted in faster resolution of sleep problems at 6 months and improvement in prosocial behavior at 12 months.	Brief behavioral sleep intervention
Koulouglioti et al., 2013 [27]	To explore the feasibility and acceptability of a home-based intervention focused on the daily sleep and feeding routines of preschoolers.	Pre-test—post-test	Mothers reported that the intervention was acceptable, and the estimated effects were in line with expectations. Personalized, home-based interventions focused on daily mealtime and sleep routines show potential for helping mothers foster and maintain healthy behaviors in their children, while also reducing the risk of overweight and obesity.	Individualized intervention
Tamura & Tanaka, 2014 [19]	To evaluate the effects of sleep education combined with a self-help behavior change strategy on sleep habits, sleep quality, and irritability in elementary school children.	Quasi-experimental	Children who received sleep education showed increased knowledge about sleep, and these gains were maintained after two weeks. The sleep education group demonstrated significant improvements in bedtime routines and total sleep duration. Positive sleep-related behaviors increased, particularly: getting up at a consistent time; avoiding screens before bedtime; not napping after school. These behavior changes were linked to reduced poor sleep and lower irritability.	Sleep education combined with self-monitoring/self-help strategies
Rigney et al., 2015 [20]	To investigate the effectiveness of a school-based sleep education program in improving key sleep behaviors, sleep knowledge, and sleep hygiene.	RCT	In general, changes in sleep patterns were small and transitory, and they had largely disappeared at follow-up.	Sleep education program
Surani et al., 2019 [15]	To assess the baseline sleep habits among elementary school children and to assess the role of media-based interventions.	Pre-test—post-test	Administering an animated video about sleep education along with a provider-based education may be an effective tool for educating elementary school students and decreasing the prevalence of these sleep-related issues.	Animated video
Fehr et al., 2016 [14]	To examine whether adding a brief cognitive-behavioral play intervention to standard parent behavior management could help reduce sleep difficulties, sleep-related anxiety, and distress in young children.	Case series	After the intervention, parents reported improvements in their children’s sleep habits, reduced sleep-related anxiety, and fewer general fears. Parents indicated high satisfaction with the treatment approach.	Brief cognitive-behavioral play intervention
Mindell et al., 2016 [25]	To examine the efficacy of Sleep Well!, a parent-based sleep education endeavor, which supplemented an outreach program that provides beds to socioeconomically disadvantaged children.	RCT ^1^	Providing beds to socioeconomically disadvantaged children resulted in increased sleep duration and decreased use of electronics at bedtime, while the combination of a bed and brief parent sleep education conferred additional sleep benefits.	Sleep education program
Ashton, 2017 [18]	To ascertain whether a universal school-based sleep education program would produce robust and sustained changes in sleep behaviors in primary-aged children.	Quasi-experimental	Although there has been a sustained increase in knowledge about sleep, there have been no statistically significant improvements in duration or behavioural efficiency.	Sleep education program
Almondes & Leonardo, 2019 [13]	To evaluate the effects of an intervention with the serious game “Perfect Bedroom: learn to sleep well” on the sleep habits of healthy children.	Experimental design	Although there was an immediate reduction in the number of electronic devices and stimulating activities in the bedroom, these changes were not maintained during the follow-up period.	Serious Game
Karimi et al., 2022 [24]	To compare the effectiveness of cognitive behavioral therapy program for insomnia, named the KiSS program and reflexotherapy on insomnia in children.	Quasi-experimental	The KiSS programme effectively reduced insomnia; however, reflexology only had an impact in the post-test and symptoms reappeared after two months.	Sleep education program; Reflexotherapy
Williamson et al., 2022 [23]	To describe the adaptation, feasibility, and initial outcomes of Sleep Well!, an intervention for early childhood insomnia and insufficient sleep.	Experimental design	There was a significant reduction in sleep problems and in the use of electronic devices in the bedroom, as well as a reduction in latency. There was also an improvement in sleep duration and cultural humility.	Sleep education program
Hassanin et al., 2023 [16]	To assess the effectiveness of sleep hygiene videos in improving sleep quality and subsequent cognitive function in children aged 10–11 years.	Experimental design	Introducing child-friendly sleep hygiene videos can improve a child’s sleep and health.	Sleep hygiene videos
Cea et al., 2024 [17]	To assess the feasibility and preliminary effectiveness of a yoga-based bedtime routine in a sample of parent–child dyads from a rural community.	RCT ^1^	The intervention was feasible, with strong adherence. More sessions resulted in improved sleep quality and reduced domestic chaos.	Yoga
Merayo et al., 2025 [21]	To assess the effectiveness of a sleep education program for young athletes in improving sleep quality, sleep duration, mood, and academic performance.	Cohort study	Sleep quality and quantity improved in athletes over 12, and school performance was enhanced across all age groups.	Sleep education program
Zhang et al., 2025 [22]	To explore the preliminary effectiveness of a program designed Sleep to Enhance Educational Performance in Schools on middle school students’ sleep-related outcomes.	RCT ^1^	In comparison with the control group, adolescents demonstrated greater improvements in sleep-related beliefs and self-efficacy, daytime sleepiness, and internalizing symptoms. Furthermore, those who initially held weaker beliefs about sleep hygiene showed the most notable gains from the program.	Sleep education program

^1^ RCT—Randomized Controlled Trial.

## Data Availability

No new data were created or analyzed in this study. Data sharing is not applicable to this article.

## Abbreviations

The following abbreviations are used in this manuscript:

ACES	Australian Centre for Education in Sleep
JBI	Joanna Briggs Institute
KiSS	Kinder mit Schlafstörungen Programm
RCT	Randomized Controlled Trial
SLEEPS	Sleep to Enhance Educational Performance in Schools
